# Supplementary material for: EbARC1, an E3 Ubiquitin Ligase Gene in Erigeron breviscapus, Confers Self-Incompatibility in Transgenic Arabidopsis thaliana
Source: Int J Mol Sci. 2020 Feb 20;21(4):1458. doi: 10.3390/ijms21041458 (PMC7073078; doi:10.3390/ijms21041458)
Supplement: Supplementary file 1 [file ijms-21-01458-s001.pdf]

Supplementary Materials

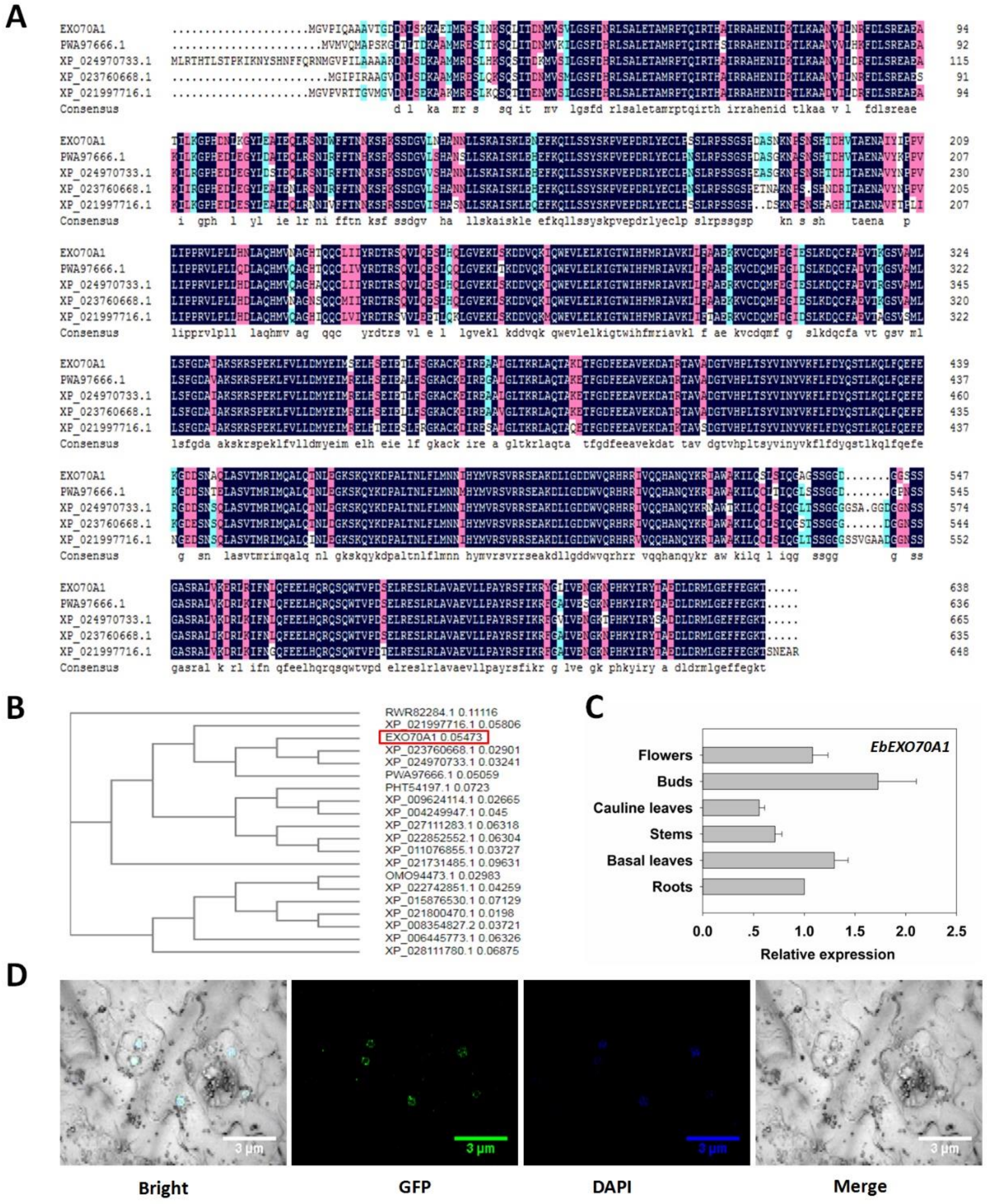

Figure S1. Analysis of bioinformation, profile expression and subcellular locization of EbExo70A1. (A) Amino acid sequence alignment of Exo70A1 proteins from *Lactuca sativ* (LsExo70A1, XP_023760668.1), *Cynara cardunculus var. scolymus* (Ccv.sExo70A1, XP_024970733.1), *Artemisia annua* (AaExo70A1, PWA97666.1), and *Helianthus annuus* (HaExo70A1, XP_021997716.1). The

conserved Exo70 domains are indicated. (B) Phylogenetic tree of EbExo70A1 and Exo70A1s from other plant species, including *Lactuca sativ* (LsExo70A1, XP_023760668.1), *Cynara cardunculus var. scolymus* (Ccv.sExo70A1, XP_024970733.1), *Artemisia annua* (AaExo70A1, PWA97666.1), *Helianthus annuus* (HaExo70A1, XP_021997716.1), *Capsicum baccatum* (CbExo70A1, PHT54197.1), *Nicotiana tomentosiformis* (NtExo70A1, XP_009624114.1), *Citrus clementina* (CcExo70A1, XP_006445773.1), *Olea europaea var. Sylvestris* (Oev.sExo70A1, XP_022852552.1), *Camellia sinensis* (CsExo70A1, XP_028111780.1), *Solanum lycopersicum* (SlExo70A1, XP_004249947.1), *Sesamum indicum* (SiExo70A1, XP_011076855.1), *Chenopodium quinoa* (CqExo70A1, XP_021731485.1), *Coffea arabica* (CaExo70A1, XP_027111283.1), *Ziziphus jujuba* (ZjExo70A1, XP_015876530.1), *Prunus avium* (PaExo70A1, XP_021800470.1), *Cinnamomum micranthum f. kanehirae* (Cmf.kExo70A1, RWR82284.1), *Corchorus olitorius* (CoExo70A1, OMO94473.1), *Durio zibethinus* (DzExo70A1, XP_022742851.1), and *Malus domestica* (MdExo70A1, XP_008354827.2). (C) Expression profile of Exo70A1 in roots, basal leaves, stems, cauline leaves, buds and flowers of *E. breviscapus.* (D) The EbExo70A1 protein was localized to the nucleus.

Supplemental table S1. Primers used in this study

Use	Constructs	Plasmid Name	Primers (5′->3′)	Cloning Metho

				d
Subcellular localization assay	EbARC1-GFP	POCA30-GFP	AAAGAGCTCATGGCATCAGCTGCTATCTTT<br>AAATCTAGATGAAACCGAAACAGACATAG	Sacl<br><br>Xbal
	EbExo70A1-GFP	POCA30-GFP	AAAGGATCCATGGGGGTTCCTATTCAAG<br>AAATCTAGAAGTCTTCCCCTCGAAAA	BamHI<br><br>Xbal
Yeast-Two Hybrid Assay	BD-EbARC1	PGBKT7	AAACATATGATGGCATCAGCTGCTATCTTT<br>AAAGGATCCCTATGAAACCGAAACAGACAT	NdeI<br><br>BamHl
	BD-EbARC(1-304)	PGBKT7	AAAGAATTCATGGCATCAGCTGCTATCTTT<br>AAAGTCGACTCCTTAGGAATGCTTACAAAAGTATC	EcoRl<br><br>Sall
	BD-EbARC(1-368)	PGBKT7	AAACATATGATGGCATCAGCTGCTATCTTT<br>AAAGGATCCCCATTGCATAATCAGATTTCT	Ndel<br><br>BamHI
	BD-EbARC(1-588)	PGBKT7	AAACATATGATGGCATCAGCTGCTATCTTT<br>AAAGGATCCTTCAGCCACATTTTCACATTCC	Ndel<br><br>BamHI
	AD-EbEXO70A1	PGADT7	AAAGAATTCATGGGGGTTCCTATTCAAGCA<br>AAACTCGAGTTAAGTCTTCCCCTCGAAAAATTC	EcoRl<br><br>Xhol
	AD-EbEXO70A1（1-284）	PGADT7	AAAGAATTCATGGGGGTTCCTATTCAAGCA<br>AAACTCGAGACGCATAAAATGAATCCATGTCC	EcoRl<br><br>Xhol
	AD-EbEXO70A1（284-639）	PGADT7	AAACATATGATTGCGGTAAAATTGTTATTTG<br>AAAGAATTCAGTCTTCCCCTCGAAAAATTCC	NdeI<br><br>EcoRl

BiFC Assay	EbARC1-nYFP	pFGC-nYFP	AAAGAGCTCATGGCATCAGCTGCTATCTTT<br>AAATCTAGATGAAACCGAAACAGACATAG	SacI<br>XbaI
	EbExo70A1-cYFP	pFGC-cYFP	AAATCTAGAATGGGGGTTCCTATTCAAG<br>AAAGGATCCAGTCTTCCCCTCGAAAA	XbaI<br>BamHI
Expression Assay	Flag-EbARC1	pOCA30	AAAGAGCTCATGGCATCAGCTGCTATCTTT<br>AAATCTAGACTATGAAACCGAAACAGACATA	SacI<br>XbaI
	Myc-EbExo70A1	pOCA30	AAATCTAGAATGGGGGTTCCTATTCAAG<br>AAAGTCGACAGTCTTCCCCTCGAAAAATTC	XbaI<br>SalI
qRT-PCR	qRT-PCR-EbARC1-F:<br>qRT-PCR-EbARC1-R:		ATTACAAGACAATGATAGCAGATGA<br>GAATGTGAAAATGTGGCTGAA	
	qRT-PCR-EbExo70A1-F:<br>qRT-PCR-EbExo70A1-R:		GATGCAAGTAATAAGAATCCTTCTA<br>TATCTTTTGGACGTCATCTTTG	
	qRT-PCR-EbACTIN-F:<br>qRT-PCR-EbACTIN-R:		GCAGGTACACACAGTCCCAT<br>GCAGGTACACACAGTCCCAT	

Predict Result					
Order	Protein Name	Locations	Score (Confidence)	Ubiquitination Sites	Substrate Motifs
1	EBEXO70A1	143	0.568765 (High)	ANNLLS K AISKLE	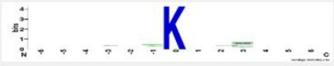
2	EBEXO70A1	147	0.568296 (High)	LSKAIS K LENEFK	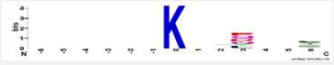
3	EBEXO70A1	258	0.581503 (High)	HQLGVE K LSKDDV	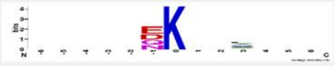
4	EBEXO70A1	261	0.689835 (High)	GVEKLS K DDVQKI	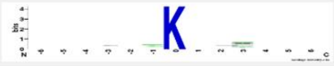
5	EBEXO70A1	266	0.664036 (High)	SKDDVQ K IQWEVL	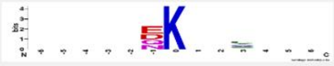
6	EBEXO70A1	333	0.567942 (High)	FGDAIA K SKRSPE	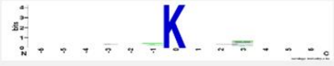
7	EBEXO70A1	335	0.504921 (High)	DAIAKS K RSPEKL	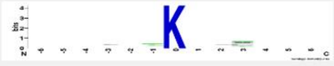
8	EBEXO70A1	340	0.729062 (High)	SKRSPE K LFVLLD	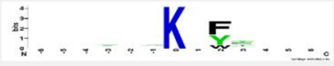
9	EBEXO70A1	379	0.674611 (High)	AALGLT K RLAQTA	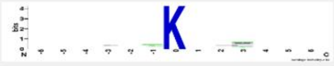
10	EBEXO70A1	386	0.51156 (High)	RLAQTA K DTFGDF	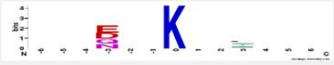
11	EBEXO70A1	497	0.513579 (High)	VRRSEA K DLLGDD	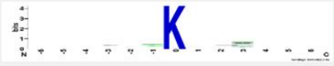
12	EBEXO70A1	613	0.572304 (High)	LLVENG K NPHKYI	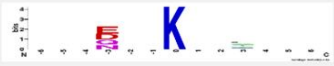

Figure S2. Prediction of EbExo70A1 ubiquitin site by UbSite. The analysis of the amino acids sequence suggested that Lys340 is a ubiquitination site, with a high score of 0.729062, where a score ≥ 0.50 (specificity ≥60%) is considered to be a positive prediction. S < 0.50 corresponds to a low confidence prediction, while a prediction with a score > 0.73 should be regarded as high confidence (specificity > 84%).
